# A Tumor-Penetrating Peptide Modification Enhances the Antitumor Activity of Thymosin Alpha 1

**DOI:** 10.1371/journal.pone.0072242

**Published:** 2013-08-19

**Authors:** Xingzhen Lao, Meng Liu, Jiao Chen, Heng Zheng

**Affiliations:** Department of Life Science and Technology, China Pharmaceutical University, Nanjing, Jiang Su Province, P.R. China; University of Helsinki, Finland

## Abstract

A serious limitation of numerous antitumor drugs is the incapacity to penetrate solid tumors. However, addition of an RGD fragment to peptide drugs might solve this problem. In this study, we explored whether the introduction of a permeability-enhancing sequence, such as iRGD (CRGDK/RGPD/EC) fragments, would enhance the activity of thymosin alpha 1 (Tα1). The modified Tα1 (Tα1-iRGD) was successfully expressed and purified, and the in vitro assay showed that Tα1-iRGD presented a similar activity as Tα1 in promoting proliferation of mouse splenocytes. Meanwhile, cell adhesion analysis revealed that Tα1-iRGD exhibited more specific and greater binding with tumor cells compared with Tα1. Furthermore, the iRGD fragment evidently enhanced the basal ability of Tα1 to inhibit proliferation of cancer cells in vitro, particularly of mouse melanoma cell line B16F10 and human lung cancer cell line H460. Our findings indicated that the addition of an iRGD fragment increased the anti-proliferative activity of Tα1 against cancer cells by improving the ability of Tα1 to penetrate the tumor cells. This study highlighted the important roles of an iRGD sequence in the therapeutic strategy of Tα1-iRGD. Thus, Tα1-iRGD could be a novel drug candidate for cancer treatment.

## Introduction

Thymosin alpha 1 (Tα1; generic drug name: thymalfasin; trade name: Zadaxin) is a 28-amino acid peptide that was first isolated from the thymus gland in mammals by Goldestein in 1977 [1]. Tα1 has already been considered as a potential agent for treating immune deficiencies and cancers for several years. Numerous experiments have confirmed the anti-proliferative activity of Tα1 against melanoma, lung cancer [2], breast cancer [3], and glioblastoma [4]. Furthermore, Tα1 activity has been evaluated in a phase II trial of patients with metastatic melanoma [5]. However, the activity of Tα1 is pleiotropic. Thus, we should find an effective way of delivering Tα1 to the target tumor cells to exert its full adjustive effect on tumor cells. During the past decades, integrins, particularly the integrin αvβ3, were proven to recognize the RGD (Arg-Gly-Asp) fragment, and αvβ3 is specifically associated with the upregulated expression of tumor vessels and certain tumor cells on the endothelium [6]. Therefore, the RGD fragment was considered a useful tool for targeting drugs to tumor [7]–[10].

The more powerful tumor-penetrating peptide iRGD (CRGDK/RGPD/EC) was identified recently based on the mechanism of RGD [11], [12]. The iRGD achieved tumor homing by initially binding to integrin αvβ3 and then being proteolytically cleaved to CRGDK/R, which can penetrate into tumor cells and tissues by binding with neuropilin-1. Therefore, we introduced iRGD to Tα1 by conjugating iRGD to the C-terminus of Tα1 with an ordinary GGGG linker. We then investigated the activity changes of the modified Tα1-iRGD. The results showed that the modified Tα1-iRGD obviously exhibited the similar or improved anti-proliferative activity against various tumor cell lines, thereby making make it a better candidate drug for specific treatment of cancers.

## Materials and Methods

### Materials

B16F10 mouse melanoma cell, human gastric cancer cell line BGC-823, human lung cancer cell line H460, and human colon cancer cells line HT-29 were available in our institute. Our institute purchased the cell lines from American Type Cell Culture (ATCC, Shanghai, China). Concanavalin A (ConA) was purchased from sigma-aldrich Company (USA). Paclitaxel (Taxol, was provided by JiangsuYew Pharmaceutical Company Limited (Wuxi, Jiangsu province of China). Enterokinase was obtained from New England Biolabs, USA. Nickel-chelating column was purchased from GE Healthcare Company. All the restriction enzymes were purchased from Takara Biotechnology (Dalian) Co., Ltd., and T_4_ DNA ligase was purchased from Promega (USA).

### Methods

#### Construction, expression, and purification of Tα1 and Tα1- iRGD

The synthetic genes coded for Tα1 and Tα1 -iRGD were digested with KpnI and HindIII and inserted into pET32a, separately. After the calcium chloride transformation, the positive clones were selected based on ampicillin resistance, which was further confirmed by DNA sequencing. *Escherichia coli* BL21 (DE3) harboring positive recombinant plasmid was grown at 37°C on Luria-Bertani medium containing 100 µg/ml of ampicillin at 37°C. Lactose (5 mM) was added to the medium for 4 h to induce protein expression, when the absorbance at 600 nm reached 0.6. Then, the cells were harvested by centrifugation (10,000 g, 10 min, and 4°C), suspended in ice-cold 10 mM Tris and 1 mM EDTA at pH 7.6, and disrupted by sonication. The supernatant of the cell lysate was further purified by a nickel-chelating affinity resin according to the manufacturer’s instruction, and the eluted fraction was analyzed by 15% sodium dodecyl sulfate-polyacrylamide gel electrophoresis (SDS-PAGE). The fusion proteins were digested by enterokinase at 23°C for 16 h. The target peptides were obtained by loading the nickel-chelating affinity column with equilibration buffer. The eluted peptides were collected, desalinated by gel filtration chromatography, and confirmed by mass spectroscopy.

#### Spleen lymphocyte proliferation assay

All experimental procedures using animals used in the our study were performed in strict accordance with the recommendations from the Guide for the Care and Use of Laboratory Animals, which is promulgated by the United States National Institute of Health, and was approved by Jiangsu Provincial Experimental Animal Management Committee under Contract 2012(su)-0035. Spleen lymphocyte proliferation experiments were performed as previously described [13]. Suspensions of single spleen cells were prepared from ICR mice (SPF, Comparative Medicine Center of Yangzhou University, China). Single cell suspension (2×10^6^/mL) was prepared in RPMI 1640 containing 10% FBS (HyClone, USA) after teasing through a sterilized autoclaved mesh. The adjusted cells were placed in a 96-well microtiter plate and added with 200 µL aliquots per well. The samples were incubated with or without recombinant Tα1 or Tα1-iRGD at 37°C in an atmosphere containing 5% CO_2_ and 95% humidity. ConA (5 µg/mL) and RPMI 1640 were set as positive and negative control, respectively. After 68 h, cells were counted by the 3-(4, 5-dimethylthiazol-2-yl)-2, 5-diphenyltetrazolium bromide (MTT) assay [14], [15]. MTT was dissolved in 5 mg/mL of PBS and filtered for sterilization. The MTT solution (20 µL) was added to each well, and the samples were cultured for another 4 h. After all the medium were removed from the wells, DMSO (150 µL) was used to solubilize the formazan crystals. The plates were placed on a plate shaker for 10 min, and then read at 570 nm using a microplate reader. The results were expressed as the relative spleen lymphocyte proliferation (%), which is calculated by the equation (A_T_ − A_N_)/A_N_ ×100% (where A_T_ refers to the absorbance of the treatment group, and A_N_ refers to the absorbance of the negative control group).

#### B16F10 melanoma cell attachment assay

Cell attachment experiments were conducted as previously described [16]. Various concentrations of recombinant Tα1 or Tα1-iRGD were coated in a 96-well ELISA plate (Costar, USA) at 4°C overnight. Then, the plates were blocked with 2% bovine serum albumin in RPMI 1640 medium at 37°C for 2 h. B16F10 melanoma cells (3 × 10^4^/100 µL) were added to the pre-coated plate. After 1h of incubation at 37°C, the plates were washed twice with isotonic buffer saline to remove unbound cells. Cells bound to the wells were fixed, stained by 0.5% crystal violet staining buffer, and photographed. Finally, 10% acetic acid (100 µL per well) was added to each well to extract the crystal violet. The remaining adherent B16F10 melanoma Cells were determined by a microplate spectrophotometer at 595 nm. The relative cell adhesion (%) was calculated using the equation (A_T_ − A_C_)/A_C_ × 100% (where A_T_ refers to the absorbance of the treatment group, and A_C_ refers to the absorbance of the negative control group).

#### Tumor cell proliferation-inhibition study in vitro

B16F10 melanoma cell in the logarithmic growth phase were dispersed in 0.25% trypsin with RPMI 1640 complete medium. Cell suspension (50 000 cells/mL) was plated into a 96- well culture plate (0.1 mL/well), which was inoculated for 8 h in 37°C. The cells were incubated with different concentrations of recombinant Tα1 or Tα1-iRGD at 37°C. Paclitaxel (0.0125 µmol/mL) was set as a positive control, whereas RPMI 1640 was set as a negative control. After 36 h, cells were counted by MTT assay [14], [15], which was described in the spleen lymphocyte proliferation assay. The results were expressed as the relative inhibition of cell proliferation (%), which is calculated by the equation (A_N_ − A_T_)/A_N_ × 100% (where A_T_ refers to the absorbance of the treatment group, and A_N_ refers to the absorbance of the negative control group). Other cells such as human gastric cancer cell line BGC-823, human lung cancer cell line H460, and human colon cancer cell line HT-29, were treated with the same method.

#### Three-Dimensional modeling

The structure of Tα1-iRGD was modeled using the DS|Built homology model (Accelrys Inc., USA) based on template (PDB file 2L9I) [17]. Then, the initial model was optimized for 2000 steps by the steepest descent minimizer and for 2000 steps by the conjugate gradient minimizer under the CHARMmH force field. The final Tα1-iRGD model was used as a ligand and docked with integrin αvβ3 (PDB file 1L5G) [18] by DS|ZDOCK [19] to determine whether Tα1-iRGD binds to αvβ3 or not. ZDOCK is the protein-protein docking software. The most probable predictions can be selected or filtered by residue conservation of the interaction sites and pose ranks.

## Results

### Cloning, Expression, and Purification of Tα1 and Tα1- iRGD

**Figure 1:**
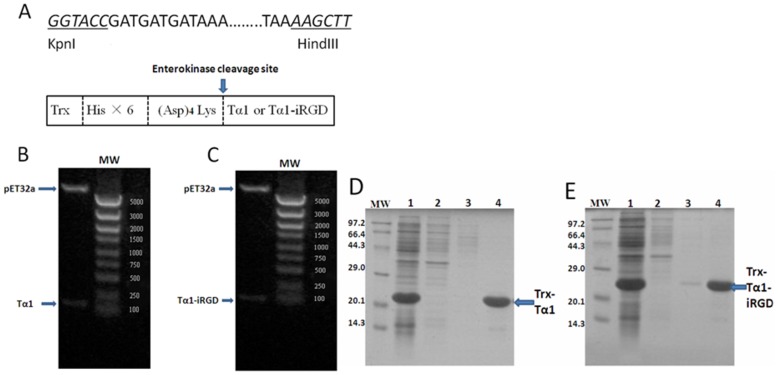
Production of Tα1 and Tα1-iRGD peptides. A. Restriction sites are indicated in underline, the translated amino acid and Trx-His×6-(Asp)_4_Lys- tag are shown in the box. B. and C., pET32a/Tα1 and pET32a/Tα1-iRGD clones digested with KpnI and HindIII and analyzed by gel electrophoresis analysis (1.2%). MW = molecular weight markers, bp; D. and E. Trx-Tα1 and Trx-Tα1-iRGD purified from BL21 (DE3) lysate, Lane 1, Trx-Tα1 or Trx-Tα1-iRGD induced by lactose in *E. coli* BL21 (DE3); Lanes 2 and 3, nonbinding protein of crude extract that were subjected to nickel-chelating affinity resin; Lane 4, purified Trx-Tα1 or Trx-Tα1-iRGD.

**Figure 2:**
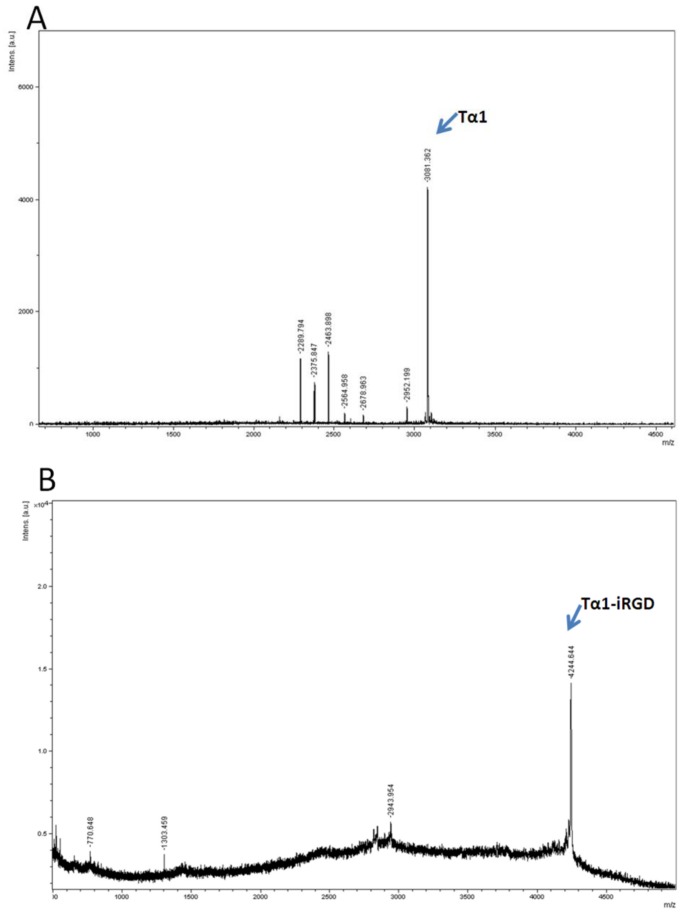
Mass spectroscopy result of recombinant Tα1 or Tα1-iRGD. A. Tα1; B. Tα1-iRGD.

Tα1 and Tα1-iRGD genes were cloned in the frame between KpnI and HindIII restriction sites of the pET32a plasmid, which produced fusion proteins partnered with Trx-A separately (Figures 1A to 1C). Most of the fusion proteins were detected as soluble proteins. The Trx fusion Tα1 and Tα1-iRGD protein was successfully induced by lactose in *E.Coli* BL21 (DE3), the fusion proteins were purified by a nickel-chelating affinity resin respectively, and then confirmed by SDS-PAGE (Figures 1D and 1E). Then, the fusion proteins were digested with enterokinase to release Tα1 and Tα1-iRGD. The molecular weight of Tα1 was detected as 3081.362 by using MALDI mass spectrometry, which is consistent with the theoretical value (Figure 2A). Meanwhile, the molecular weight of Tα1-iRGD was 4244.644, which is in agreement with the expected value as well (Figure 2B). These results confirm the successful expression of soluble Tα1 and Tα1-iRGD.

### Tα1-iRGD Showed Similar Spleen Lymphocyte Proliferation Activity with Tα1 in vitro

**Figure 3:**
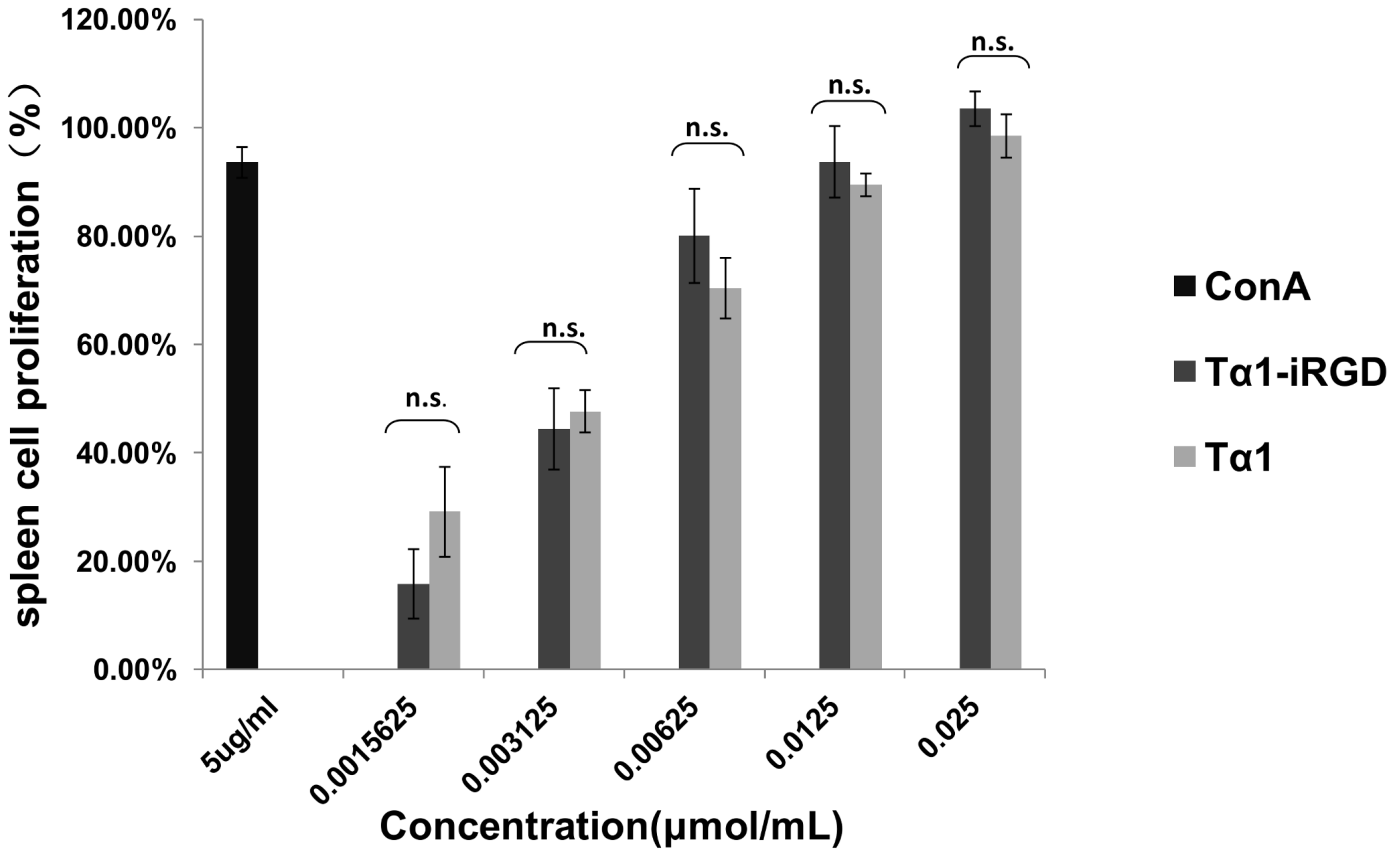
Tα1-iRGD and Tα1 showed similar spleen lymphocyte proliferation activity. Spleen lymphocytes were treated with Tα1-iRGD and Tα1. ConA was used as positive control. All data are expressed as the mean ± standard deviation, n = 4. Statistical analyses were performed with Student's t-test, n.s., not significant; *p<0.05; **p<0.01; ***p<0.001.

Different concentrations of recombinant Tα1 or Tα1-iRGD were added to spleen lymphocyte to test their proliferation activity. As shown in Figure 3, recombinant Tα1 and Tα1-iRGD shared extremely similar activities in promoting the proliferation of mouse spleen cells. In addition, a significant dose-dependent proliferation of spleen lymphocyte can be observed in Figure 3. The spleen lymphocyte proliferation assays confirmed that similar to Tα1, Tα1-iRGD has the capability to stimulate the proliferation of spleen lymphocyte, indicating that the conjugation of iRGD to the C- terminus of Tα1 does not change its spleen cell proliferation activity.

### Tα1-iRGD Increased B16F10 Melanoma Cell Attachment in vitro

**Figure 4:**
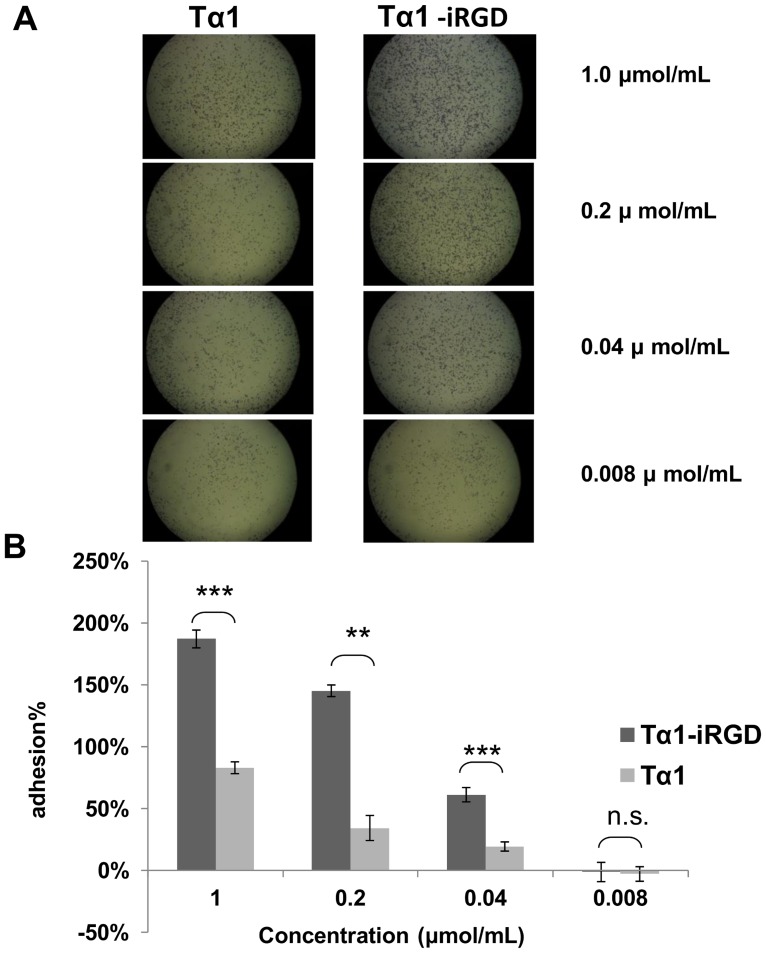
Enhanced cell attachment effect by adding iRGD to the C-terminus of Tα1. A. First, cells bound to the wells were fixed, stained by 0.5% crystal violet staining buffer and photographed. B. Second, crystal violet was extracted and the remaining adherent B16F10 Melanoma Cells were recorded by a microplate spectrophotometer at 595 nm wavelengths. All data are expressed as the mean ± standard deviation, n = 3. Statistical analyses were performed with Student's t-test, n.s., not significant; *p<0.05; **p<0.01; ***p<0.001.

The cell attachment of Tα1-iRGD and Tα1 to a melanoma cell line was further evaluated to determine whether the addition of the iRGD motif to Tα1 enhances its binding to the tumor cell or not. PBS wells were used as negative control. As shown in Figures 4A and 4B, both Tα1-iRGD and Tα1 showed a significant dose-dependent attachment to melanoma cells in this assay. However, Tα1-iRGD specifically enhanced the attachment effect to cancer cells, particularly at a concentration of 1.0 µmol/mL or at an even more low concentration of 0.04 µmol/mL. For instance, at concentrations that ranged from 0.008 µmol/mL to 1.0 µmol/mL, Tα1-iRGD exhibited significantly higher activity than Tα1 (p value <0.01) in three out of the four activities compared, particularly at 0.04 and 1.0 µmol/mL, This finding indicates that Tα1-iRGD can attach to B16F10 melanoma cell line more effectively (p value <0.01).

### Tα1-iRGD Increased the Inhibition of Tumor Cell Proliferation in vitro

**Figure 5:**
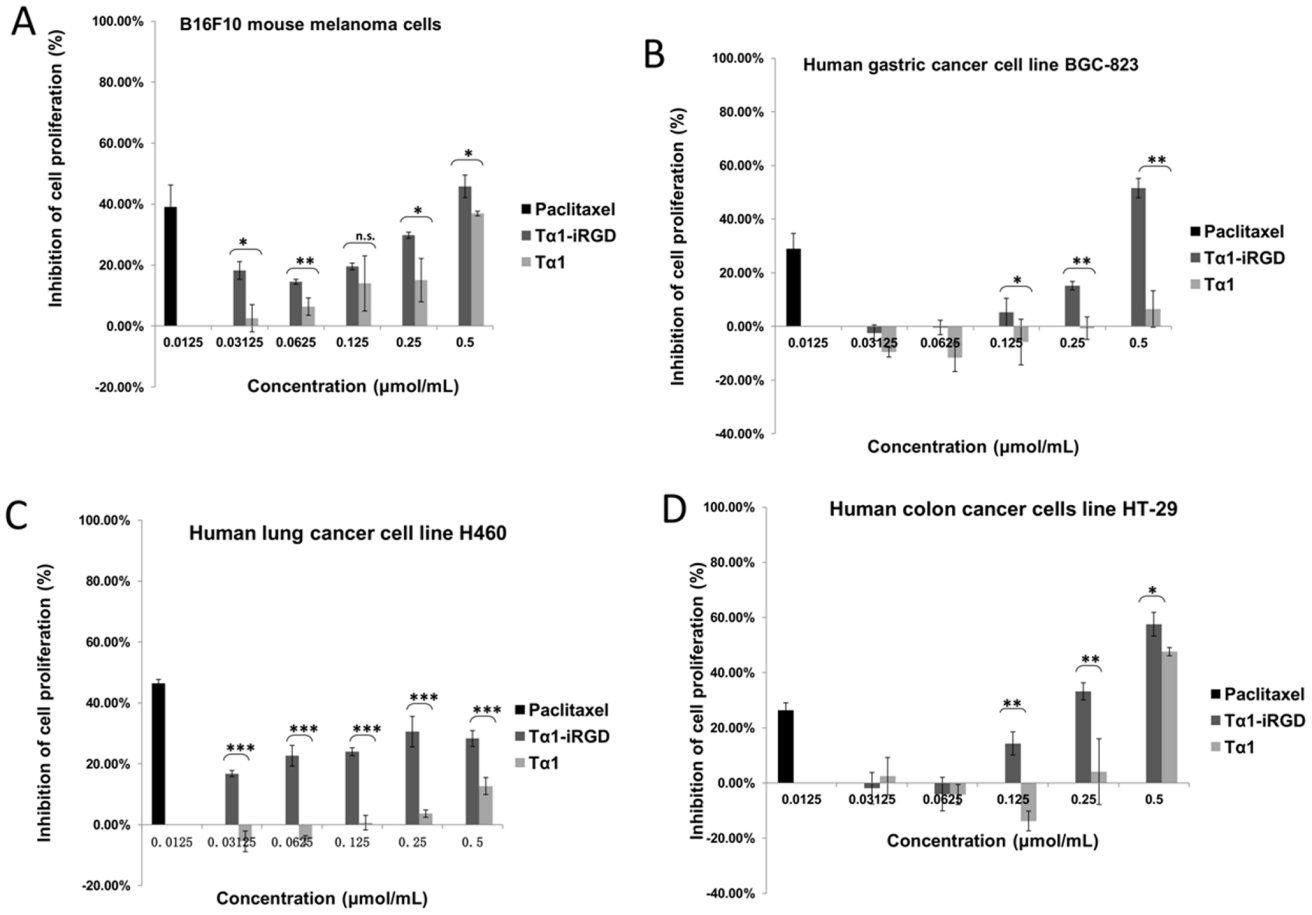
Inhibition of cancer cell proliferation by Tα1 or Tα1-iRGD. Tα1 or Tα1-iRGD was added to the cell suspension and incubated for 36 h and counted by MTT assay. Paclitaxel (0.0125 µmol/mL) was set as a positive control. RPMI 1640 was set as a negative control. All data were expressed as the mean ± standard deviation, n = 4. Statistical analyses were performed with Student's t-test, n.s., not significant; *p<0.05; **p<0.01; ***p<0.001.

Tumor cell proliferation was analyzed by MTT assay to determine whether the modification of Tα1 affected its biological activity. Figure 5 indicates that the addition of iRGD to the C-terminus of Tα1 generally enhanced its basal antiproliferative activity in general. As shown in Figure 5A,cell growth was inhibited in a dose-dependent manner when the B16F10 melanoma cells were treated with various doses of Tα1 or Tα1-iRGD (0.03125 µmol/mL to 0.5000 µmol/mL). However, Tα1-iRGD exhibited significantly higher antiproliferative activity than Tα1 at concentrations of 0.03125, 0.0625 and 0.2500 µmol/mL. These results indicated that the conjugation of iRGD to the C-terminus of Tα1 might increase its basal antiproliferative activity against the melanoma cell line B16F10.

When human gastric cancer cell line BGC-823 was treated with Tα1 or Tα1-iRGD at doses that ranged from 0.03125 µmol/mL to 0.5000 µmol/mL (Figure 5B), Tα1 exhibited no antiproliferative activity even at high concentrations (beyond 0.25 µmol/mL), By contrast, Tα1-iRGD exhibited a significant proliferative activity at high concentrations (from 0.125 µmol/mL and above).This finding indicated that the addition of the iRGD motif can improve the antiproliferative activity of Tα1 in the human gastric cancer cell line BGC-823.

Furthermore, when human lung cancer cell line H460 was treated with Tα1 or Tα1-iRGD at doses that ranged from 0.03125 µmol/mL to 0.5000 µmol/mL (Figure 5C), Tα1-iRGD exhibited significantly higher antiproliferative activity than Tα1 (p value <0.001). Tα1 only exhibited a slight cell growth inhibition activity at high concentrations (from 0.2500 µmol/mL to 0.5000 µmol/mL). By contrast, Tα1-iRGD exhibited significant antiproliferative activity even at very low concentrations. For instance, Tα1-iRGD inhibited human lung cancer cell line H460 proliferation by 16.8% at 0.03125 µmol/mL, whereas Tα1 had no antiproliferative activity at the same concentration. At 0.125 µmol/mL, Tα1-iRGD inhibited human lung cancer cell line H460 proliferation by 24.0%, whereas Tα1 only had 0.64% inhibition.

Moreover, when human colon cancer cell line HT-29 was treated with Tα1 or Tα1-iRGD at doses that ranged from 0.03125 µmol/mL to 0.5000 µmol/mL (Figure 5D), Tα1-iRGD also exhibited significantly higher antiproliferative activity than Tα1. For instance, Tα1 exhibited cell growth inhibition activity at 0.5000 µmol/mL, whereas Tα1-iRGD exhibited a significant antiproliferative activity even at low concentrations. At 0.1250 µmol/mL, Tα1-iRGD inhibited human colon cancer cell line HT-29 proliferation by 14.3%, whereas Tα1 had no antiproliferative activity. These results indicated that the addition of the iRGD fragment increased the antiproliferative activity of Tα1 against cancer cells.

### Three-Dimensional Modeling

**Figure 6:**
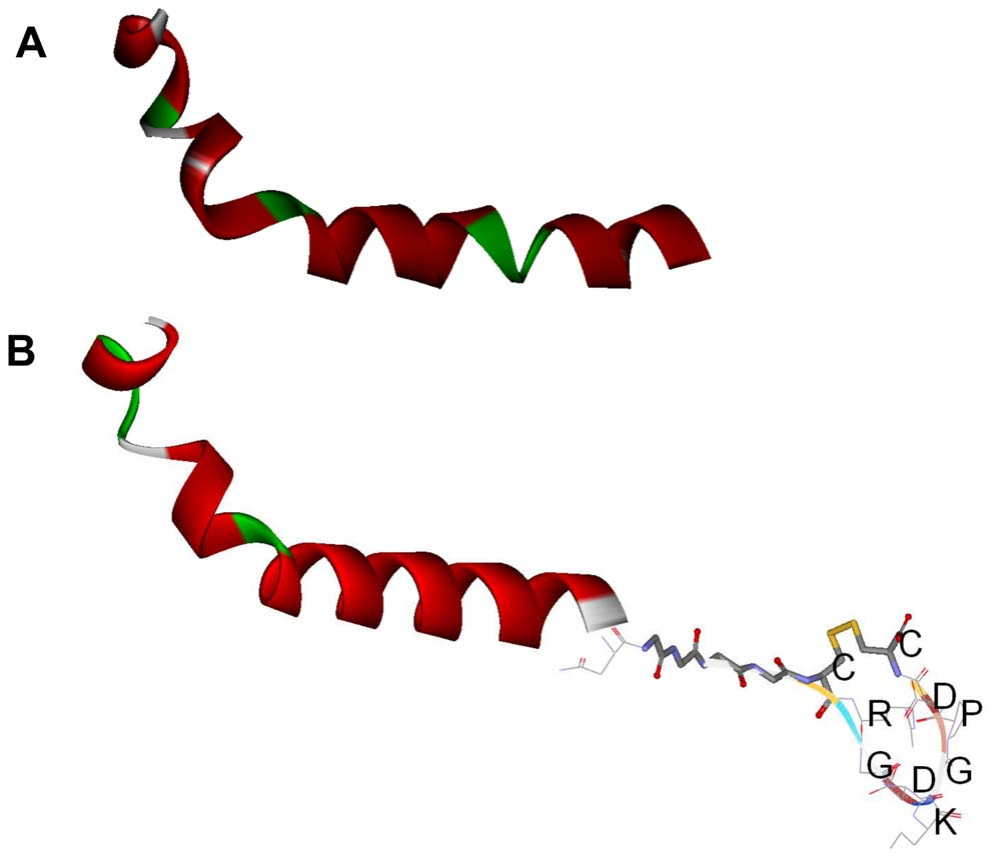
Modeling of the structures of the composed peptides by DS|Built homology model. A. Tα1 B. Tα1-iRGD.

**Figure 7:**
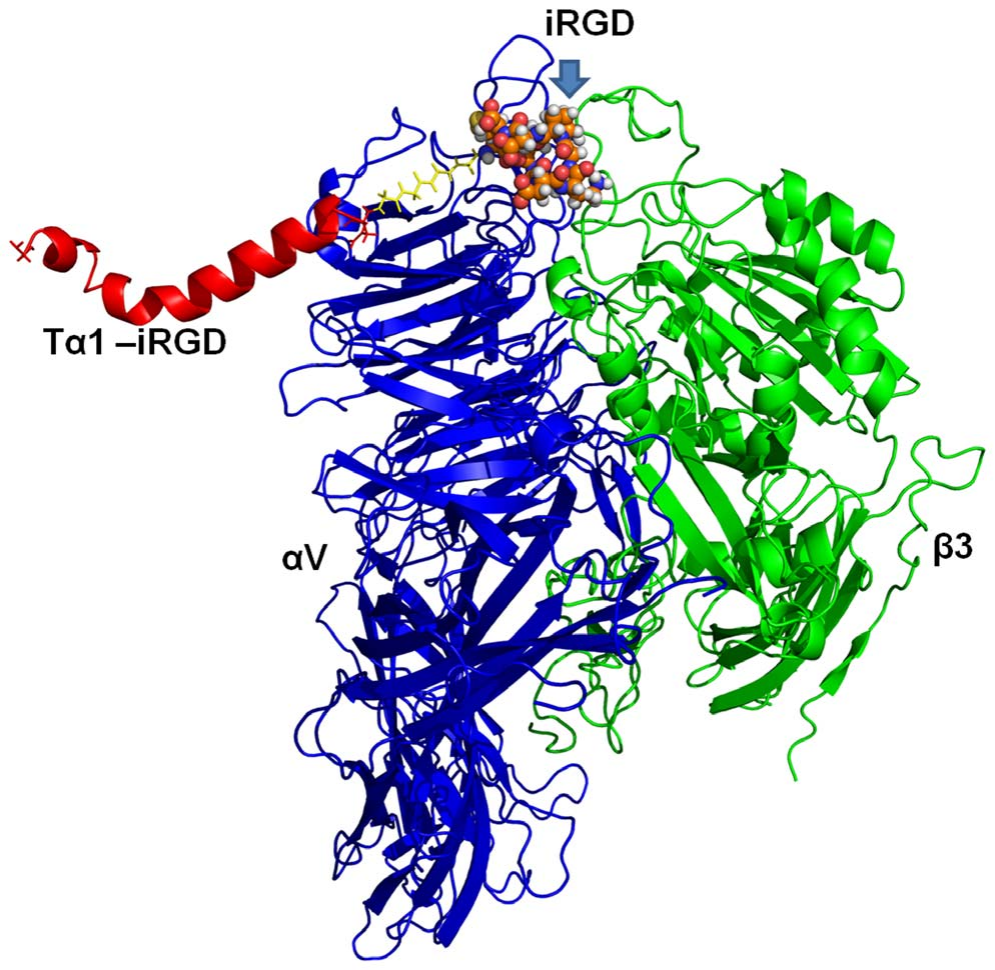
Structure of integrin αvβ3 complexed with Tα1-iRGD. The structure of αv and β3 subunits, respectively, in a ribbon-like structure. The Tα1 part of Tα1-iRGD is shown in red, the iRGD part is shown as a spherical structure, and the linker between them is shown in yellow. The αvβ3-Tα1-iRGD structure reveals that the iRGD of Tα1-iRGD inserts into a crevice between αv and β3 on the integrin head, sharing a similar interaction manner with the structure of αvβ3 in complex with a cyclic RGD ligand (PDB file code:1L5G).

The 3D structures of Tα1 and Tα1-iRGD were compared using computational modeling to determine how the addition of the iRGD motif improves the efficacy of Tα1 to arrest tumor cell growth. However, no obvious differences can be observed from the modeling (Figure 6A and 6B). Although the modeling method might not totally reflect the subtle changes of Tα1 structure due to computational ability, this result gave an implication that the iRGD might not directly affect the structure significantly. Based on previous studies on iRGD [11], [12], we assumed that the main reason of the better antiproliferative activity against tumor cells of Tα1-iRGD than Tα1 is attributed to the ability of iRGD to facilitate the more effective targeting and attachment of Tα1 to tumor cells by recognizing integrins. Thus, we further investigated the interaction between Tα1-iRGD and integrin αvβ3 using computational modeling approach. The 3D structure of integrin αvβ3 (PDB file 1L5G) was used as the receptor model in our dock research. The 1L5G describes a crystal structure of the outside membrane segment of integrin αvβ3 binding with a cyclic pentapeptide ligand that contains the Arg-Gly-Asp sequence. The structure revealed that the cyclic pentapeptide binds at the head interface between the αv and the β3 subunits, and the main contact area with the integrin involves Asp150, Gln180, and Asp218 in the αv subunit; and Ser121, Ser123, Asn215, Asp217, and Ala 218 in the β3 subunit. The iRGD part of Tα1 -iRGD was found capable of inserting into a groove between αv and β3 on the integrin head in our dock model (Figure 7), sharing a similar interaction manner with the structure of αvβ3 of an RGD ligand. The contacts between the iRGD part and the αv subunit primarily involve Asp150, whereas the contacts between the iRGD part and the β3 subunit primarily involve Ser121, Ser123, and Ala 218. Here, the computational model may have limitations to reflect real protein-ligand interaction in detail. However, it is important to illustrate the phenomenon observed from experiments and understand the potential mechanism of the iRGD motif. Therefore, our model revealed that the adding iRGD to the Tα1 might have enabled the new function of binding to αvβ3, resulting in tumor-homing. However, both Tα1 and the Tα1 part in Tα1-iRGD shared the same helical structure in our models. Thus, the similarity between the structures of Tα1 and Tα1-iRGD might also explain why Tα1 and Tα1-iRGD exhibited similar spleen lymphocyte proliferation capability.

## Discussion

Tα1 has already been approved for the treatment of Hepatitis B and C in several countries [20], [21].Clinically, Tα1 has been clinically proven to exert an immune modulatory activity on maturation of T cell [22], [23] and the natural killing cell and activation of dendritic cells [24] and the up-regulation of high affinity interleukin-2 receptors [25]. However, the effect of Tα1 is pleiotropic. Garaci E et al. recently reported that Tα1 is capable of increasing the expression of major histocompatibility complex class I surface molecules and tumor antigens in murine and human tumor cell lines [26]. These effects represent the potential factors for increasing the antitumor activity of Tα1. The therapeutic use of the Tα1 in human melanoma is currently on phase II trial. Although the antitumor activity exhibited by Tα1 has numerous benefits, the pleiotropic effect of Tα1 decreases the specificity of the treatments. Therefore, Tα1 also has relatively adverse effects on healthy cells similar to other anti-cancer drugs. Furthermore, the therapeutic efficacy and specificity of Tα1 is expected to increase by improving its capability to penetrate tumor cells. Given the fact that the expression or elevation of integrin αvβ3 and neuropilin-1 are only restricted to tumors [9], [27], [28], we propose a strategy to improve the targeted delivery of Tα1 to tumor cells by adding the iRGD fragment to the C-terminus of Tα1. The motif of iRGD is a tumor-homing peptide that has a high affinity partnering with integrin αvβ3, which has been proven to remarkably enhance the activity of antitumor drugs [12].

In this study, both Tα1 and Tα1-iRGD were successfully expressed and purified by bioengineering methods in *E. coli*. Cell attachment assay, spleen cell proliferation experiments and tumor cell proliferation experiments revealed that Tα1-iRGD has higher antitumor activity than Tα1. In the melanoma cell attachment assay, we found that Tα1-iRGD has higher attachment activity than Tα1. In the cancer cell inhibition assay, we also found that Tα1-iRGD has a better anti-proliferation activity in several cancer cell lines than Tα1, particularly in mouse melanoma cell line B16F10 and human lung cancer cell line H460. With the aid of the iRGD, Tα1-iRGD directly inhibited the proliferation of human lung cancer cell line H460 and mouse melanoma cells line B16F10 at very low concentrations in contrast to the slight inhibition exhibited by Tα1.

Previous publications have reported the effectiveness of Tα1 in clinical trials of non-small cell lung cancer [29], [30] and advanced metastatic melanoma patients [31], [32]. Our experiment showed that Tα1-iRGD can inhibit the growth of mouse melanoma cell line B16F10 and human lung cancer cell line H460 more effectively. Thus, Tα1-iRGD can be an attractive strategy for a novel therapeutics against neoplastic diseases. The positive result of cell attachment assay might be able to explain the better anti-proliferation activity in cancer cell lines of Tα1-iRGD than Tα1. These results can be further explained by our computational model. Although the computational model may have limitations to represent the real protein structure and protein-ligand interactions in detail since it merely basing on the integrations of desolvation, shape complementarity and electrostatics [19]. However, it is very helpful to understand the potential mechanism of how iRGD motif affects the function of Tα1. In our model, the iRGD part of Tα1-iRGD can form a stable complex with integrin αvβ3 in our model resulting in the tumor cell homing. In summary, the addition of iRGD significantly improved the antitumor effect of Tα1. However, the immune modulatory activity of Tα1-iRGD is almost the same with that of Tα1.

## Conclusion

In this study, we tried to connect Tα1 and tumor-homing. We expressed and evaluated the effectiveness of Tα1- iRGD in vitro for the first time. The research on Tα1- iRGD fusion peptide provided a new effective way of maximizing the effectiveness of Tα1 in treating solid tumors. The iRGD Tα1 fusion peptide could be an attractive strategy for designing novel therapeutics against tumors. We are currently studying the activity of Tα1- iRGD on cancer targeting, and the pharmacokinetic and pharmacodynamic aspects of Tα1- iRGD will be further investigated to guarantee better clinical application.
